# *Deqi* Induction by HT7 Acupuncture Alters Theta and Alpha Band Coherence in Human Healthy Subjects

**DOI:** 10.1155/2017/7107136

**Published:** 2017-04-06

**Authors:** Go-Eun Lee, Jong-Min Yun, Seung-Bum Yang, Yeonseok Kang, Hyung-Won Kang, Kwang-Ho Choi, Junbeom Kim, O. Sang Kwon, Ji-Eun Park, Jae-Hyo Kim

**Affiliations:** ^1^Department of Oriental Rehabilitation Medicine, Korean National Rehabilitation Center, Seoul, Republic of Korea; ^2^Department of Internal Medicine, College of Korean Medicine, Wonkwang University, Iksan, Republic of Korea; ^3^Department of Meridian & Acupoint, College of Korean Medicine, Wonkwang University, Iksan, Republic of Korea; ^4^Department of Medical Non-Commissioned Officer, Wonkwang Health Science University, Iksan, Republic of Korea; ^5^Department of Medical History, College of Korean Medicine, Wonkwang University, Iksan, Republic of Korea; ^6^Department of Korean Neuropsychiatry Medicine, Wonkwang University Sanbon Hospital, Gunpo, Republic of Korea; ^7^Korea Institute of Oriental Medicine, Daejeon, Republic of Korea

## Abstract

The aim of this preliminary study is to investigate the changes in phase synchronization in the theta and alpha bands before and during the performance of classical acupuncture on the Sinmun (HT7). The electroencephalogram (EEG) signals from nine healthy young subjects were recorded before and during acupuncture in the “closed-eye” state. The EEG signals were acquired from 19 surface scalp electrodes (FP1, FP2, F7, F3, Fz F4, F8, T3, C3, Cz, C4, T4, T5, P3, Pz, P4, T6, O1, and O2). Needles were inserted into the HT7 bilaterally and were then manipulated to induce* deqi* and retained for 15 minutes. Phase synchronization was measured by phase coherence. In the theta band, coherence significantly increased between the temporal (T5, T6) and occipital areas (O1, O2) during the acupuncture stimulation. In the alpha band, coherence significantly increased between the left temporal area (T5) and other areas (frontal, parietal, and occipital). Phase coherence in the theta and alpha bands tended to increase during the retention of the acupuncture needles after* deqi*. Therefore, it can be concluded that acupuncture stimulation with* deqi* is clinically effective via the central nervous system (CNS).

## 1. Introduction

Acupuncture is a major part of the traditional Korean medicine treatment and has been used for the effective clinical treatment of many diseases. The traditional Korean medicine theories state that a needle stimulates meridian points, generating qi (vital energy) and allowing qi to reach a particular organ. Thus,* deqi* is closely related to the effectiveness of the acupuncture treatment. Furthermore, previous clinical studies have reported that a needle stimulus accompanied by* deqi* is more effective than a stimulus without* deqi *[1–3].

The nature of the* deqi* mechanism has not been clearly established yet, although some studies on neurological image changes claim to have identified the neurophysiological mechanism of* deqi*. A previous study using fMRI has reported that when qi is acquired, the limbic/subcortical structure is observed to be inactivated, while various motor cortices are activated [4–8]. Therefore, the possibility was raised that a needle stimulus can adjust the limbic-paralimbic-neocortical network and serve as a mediator for analgesia, anxiolytic, and relief from various diseases.

However, most previous studies have investigated the change of power in each frequency band using power spectral analysis, while almost no research has been conducted on functional connectivity changes. In previous studies using the power spectral analysis, the results were inconsistent. The frequency band the power of which was increasing varied over previous studies, such as the theta band [9], the alpha band [10, 11], the low-frequency band (except gamma) [12], and the all-frequency band [13].

Oscillatory activity based on scalp electroencephalogram (EEG) reflects the intra- and interregional interactions of the brain [14, 15]. Among these interactions, oscillations of the theta frequency band are linked to the integration of sensory information and motion output [16]. In particular, oscillation of the theta frequency band involves participation in pain perception [17, 18] and other perceptual tasks [19]. Additionally, theta oscillations were found to be related to the induction of synaptic plasticity, spatial learning, and behavioral memory [20]. Furthermore, alpha oscillations were found to be related to selective attention, retention period of memory processing, and default mode [21–23].

Coherence is one of the indicators to measure the functional connectivity and is very sensitive to profiling of a mental state [24, 25]. Therefore, in various studies, coherence has been used to investigate the change of brain activity during performing cognitive tasks [26, 27] and meditation [28–30]. In addition, theta coherence represents a neural index of readiness to perceive and integrate sensory inputs [20, 31]. However, only one study observed the change of theta coherence during acupuncture stimulation [32].

In this context, the present study seeks to observe patterns in the changes of coherence of the theta and alpha frequency bands to disclose the effects of a needle stimulus on the functional connectivity of the cerebral cortex.

## 2. Methods

### 2.1. Participants

The study participants were 16 individuals aged 19–35 years who met the following inclusion criteria: they (1) do not major in the traditional Korean medicine; (2) do not consume cigarettes, tea, or coffee and display normal sleeping and dieting patterns on the experimental day; (3) are right-handed; and (4) have received an explanation of the purpose and process of this clinical study and have agreed to participate voluntarily.

The exclusion criteria were as follows: (1) acupuncture treatment in the 3 months prior to registration; (2) a serious physical disease or a mental disorder (based on a clinical laboratory examination, including electrocardiogram, chest X-ray, and vital signs, by the clinical study manager or doctors who assess the volunteers); (3) congenital diseases, psychiatric disorders, CNS diseases, peripheral nervous system diseases, endocrine system diseases, immunological diseases, and/or serious heart, liver, or renal problems; (4) neurological diseases, including head trauma and mental disorders, such as major depression, anxiety disorders, bipolar disorders, and schizophrenia; (5) a pacemaker, brain stimulus device, ventriculoperitoneal shunt, or other intracranial devices; (6) taking medications, except for vitamins (e.g., beta blockers, aspirin, nonsteroidal anti-inflammatory drugs, steroids, phenothiazines, selective serotonin reuptake inhibitors, statins, and angiotensin converting enzyme inhibitors) within 1 week after registering for the clinical study; (7) consumption of food containing caffeine (including coffee milk), black tea, green tea, cocoa, cola, chocolate, or caffeine-rich energy drinks within 2 hours before the clinical trial; (8) fear of acupuncture treatment or unsuitability for acupuncture treatment due to bleeding or blood coagulation diseases; (9) pregnancy or nursing a baby; (10) participation in other clinical studies in the past month or current participation in a different clinical study; and (11) unsuitability for a clinical trial as determined by the clinical study staff or the clinical study manager.

### 2.2. Acupuncture Treatment

Agreement from the subjects to participate in the clinical study and the approval of the Traditional Korean Medicine Hospital IRB of Wonkwang University in Iksan, Republic of Korea (WKUIOMH-IRB-2015-01), were obtained. Acupuncture was applied at Sinmun (HT7) bilaterally by the traditional Korean medicine doctors with over ten years of experience of providing acupuncture treatment. Comfortably seated in a chair, with their eyes closed, the participants received acupuncture stimulation. A stainless steel needle of the diameter of 0.25 mm and the length of 30 mm was inserted into the HT7 perpendicularly with the depth of 10 ± 2 mm. To obtain the* deqi* sensation without inflicting harmful pain, we readjusted the needle position whenever the participants complained of a sharp pain and then observed that the sharp pain disappeared in several seconds. After obtaining the* deqi*, the traditional Korean medical doctor stimulated the HT7 by turning the needle 180 degrees once per second for 10 seconds. This procedure was repeated three times every five minutes with a needle retention time of 15 minutes.

### 2.3. Electrophysiological Recording

The resting EEG in the closed-eye condition was measured for 10 minutes (5 minutes before acupuncture and 5 minutes during the retention of the acupuncture needle). The participants were instructed to remain still and stay relaxed, but to remain alert with their eyes closed while moving their eyes or bodies as little as possible during the recording periods. The EEG was measured with 19 electrodes according to the extended international 10–20 system with linked ears reference. A ground lead was placed between the Fz and Cz. All electrode impedances were below 5 kΩ. The EEGs were recorded using a Mitsar-EEG 201 machine (Mitsar, Russia) with a 16-bit ADC (analog to digital conversion) at the sampling rate of 128 Hz. A high-frequency filter was set to 70 Hz, a notch filter was set to 55–65 Hz, and a low-frequency filter was set to 0.3 Hz.

### 2.4. Method of the EEG Data Analysis

The EEG signals were imported into the NeuroGuide software (NeuroGuide 2.8.8, Applied Neuroscience, Inc., USA). This device has 510(k) clearance by the FDA. The removal of artifacts and the calculation of the statistical properties of the segments were performed using the NeuroGuide software. Artifacts were removed both by automatic algorithms in the NeuroGuide software and through visual inspection. Both split-half and test-retest reliability tests were conducted on the edited, artifact-free EEG segments. Only records with >95% split-half reliability, >90% test-retest reliability, and a total measurement of over 1 minute were used for further analyses.

Fast Fourier transform (FFT) autospectral and cross-spectral analyses were computed at 2 s epochs, thus yielding a 0.5 Hz frequency resolution over the frequency range of 0 to 30 Hz for each epoch. The 75% sliding window method [33] was used to compute the FFT, where successive 2 s epochs (i.e., 256 points) were overlapped by 500 ms steps (64 points) to minimize the effects of the FFT windowing procedure. Absolute power and relative power were computed from the 19 scalp locations in the theta (4.0–7.5 Hz) and alpha (8–12 Hz) frequency bands. The EEG coherence and phase were computed for all 171 intrahemispheric and interhemispheric pairwise combinations of electrodes [34]. Coherence was defined as follows:

(1) $$\begin{array}{c} \Gamma_{xy}^{2}\left(\;f\;\right)=\frac{\left(G_{xy}{\left(f\right)}^{2}\right)}{\left(G_{xx}\left(f\right)G_{yy}\left(f\right)\right)}, \end{array}$$

where *G*_*xy*_(*f*) is the cross-power spectral density and *G*_*xx*_(*f*) and *G*_*yy*_(*f*) are the respective auto-power spectral densities. Coherence was computed for all pairwise combinations of the 19 channels for the theta and alpha bands. The computational procedure for obtaining coherence involved computing the power spectra for *x* and *y* and then computing the normalized cross-spectra. As complex analyses were involved, this resulted in the production of the cospectrum (“*r*” for real) and quad-spectrum (“*q*” for imaginary). Coherence was then computed as follows:

(2) $$\begin{array}{c} \Gamma_{xy}^{2}=\frac{r_{xy}^{2}+q_{xy}^{2}}{G_{xx}G_{yy}}. \end{array}$$

### 2.5. Measurement of* Deqi* Sensation

Using the Southampton acupuncture sensation questionnaire [35],* deqi* sensations felt by the participants during the acupuncture treatment were measured. Three (pricking, sharp, and electric shock) of the 17 sensations measured were irrelevant to* deqi*. The remaining 14 measured sensations relevant to* deqi* were classified to 2 dimensions. The first dimension, which concerns* deqi* related to aching sensations, includes 7 sensations (deep ache, dull ache, uncomfortable, heavy, pressure, bruised, and stinging) closely related to pain. The second dimension, which concerns* deqi* related to tingling sensations, includes 7 sensations (tingling, warm, spreading, fading, numb, twinge, and throbbing) that are less related to pain.

### 2.6. Statistical Method

Statistical analyses were performed using SPSS 22.0 (IBM Corp., Armonk, NY, USA). The paired* t*-test was applied to compare the coherence data before acupuncture and during the acupuncture needle retention time. The alpha value for significance was set to 0.05.

## 3. Results

### 3.1. Participant Characteristics and* Deqi* Sensations

A total of 16 healthy participants were sampled, two of whom were excluded for their own personal reasons, and 14 remaining subjects underwent EEG and participated in the acupuncture treatment. The participants were all right-handed and showed no neurological symptoms or evidence of nervous system diseases or psychiatric disorders. The clinical study was conducted in Iksan, South Korea, between November and December of 2015.

**Figure 1:**
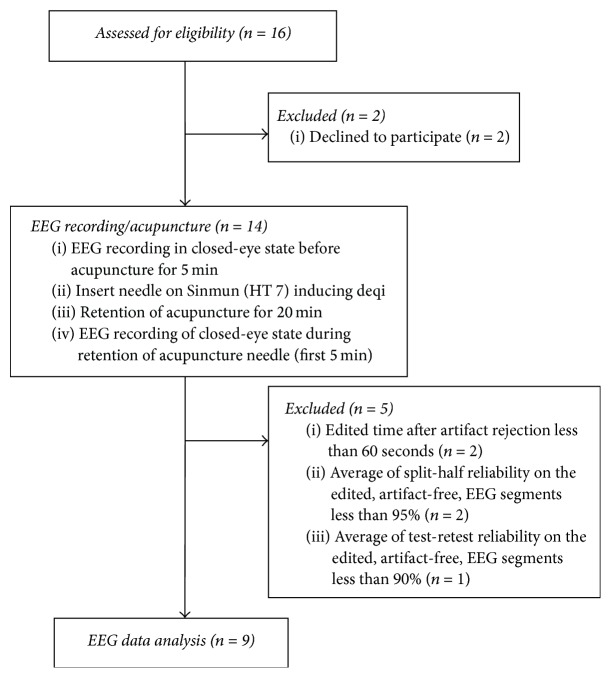
Flow diagram of study participants.

To ensure an accurate analysis of the EEG data, after the removal of artifacts, we excluded 2 participants with the edit time of under 60 seconds, 2 participants with the average split-half reliability under 95%, and 1 participant with the average test-retest reliability under 90% (see Figure 1). A total of 9 participants (5 males and 4 females) of the average age of 21.33 ± 2.18 years were included in the analysis of the EEG data. Evaluation of these 9 participants resulted in a total* deqi* score of 12 points with the average aching dimension* deqi* score of 6.56 points and the average tingling dimension* deqi *score of 5.44 points (see Table 1).

### 3.2. Coherence

**Figure 2:**
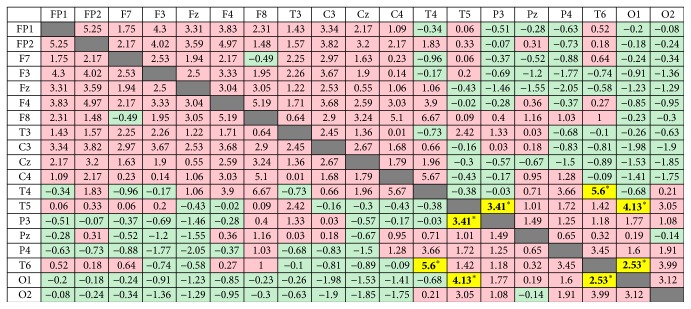
Coherence changes in the theta band before and during HT7 acupuncture. Data show the difference between mean coherence during retention of the acupuncture needle and mean coherence before acupuncture. The green areas represent a decrease of coherence during the acupuncture treatment. The red areas represent an increase of coherence during the acupuncture treatment. Significantly increasing areas are marked with the yellow color (^*∗*^*p* < 0.05).

The value of coherence on each electrode is presented in detail in Supplement  1 (see Supplementary Material available online at https://doi.org/10.1155/2017/7107136). Theta coherence increased significantly after* deqi* during the retention of the acupuncture needles at T4-T6, T5-P3, T5-O1, and T6-O2. Albeit insignificant, theta coherence tended to increase inside the frontal region and in the frontal and central regions (see Figure 2).

**Figure 3:**
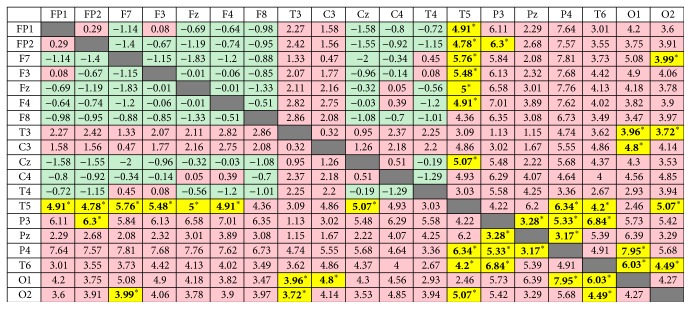
Coherence changes in the alpha band before and during HT7 acupuncture. Data show the difference between mean coherence during retention of the acupuncture needle and mean coherence before acupuncture. The green areas represented a decrease of coherence during the acupuncture treatment. The red areas mark an increase of coherence during the acupuncture treatment. Significantly increasing areas are marked with the yellow color (^*∗*^*p* < 0.05).

Alpha coherence increased significantly at FP1-T5, FP2-T5, FP2-P3, F7-T5, F7-O2, F3-T5, Fz-T5, F4-T5, T3-O1, T3-O2, C3-O1, Cz-T5, T5-P4, T5-T6, T5-O2, P3-Pz, P3-P4, P3-T6, Pz-P4, P4-O1, T6-O1, and T6-O2 during the retention of the acupuncture needles after* deqi* (see Figure 3). Furthermore, albeit not significant, alpha coherence tended to increase between the frontal-parietal and frontal-occipital regions (see Figure 3).

**Figure 4:**
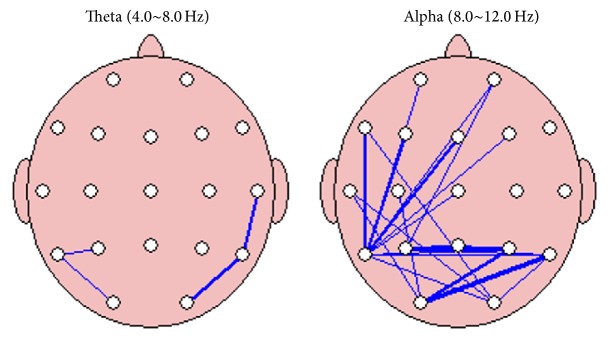
Brain mapping showing significant statistical changes of theta and alpha coherence. Blue hair line means significance with *p* < 0.05 and blue thick line means significance with *p* < 0.01.

The links between the nodes indicate a significant change (*p* < 0.05) in synchronization between the two channels before acupuncture and during the retention of the acupuncture needles. The blue color lines indicate the strength of the coherence during acupuncture stimulation (see Figure 4).

## 4. Discussion

Acupuncture treatment has been widely used to treat various diseases. Notably, acupuncture treatment has been considered as a minimally invasive and safe treatment for high-risk patients, alleviating pain with a low risk of complications [36]. Additionally, acupuncture treatment has been reported to relieve mental fatigue [37, 38]. Among the meridians, the heart meridian is known to be deeply related to the brain and mental function. In addition, HT7, the acupoint of the heart meridian, is intensively used in clinical procedures to treat insomnia, epilepsy, mental fatigue, anxiety, and other psychiatric disorders and is known to be safe and effective.

Among the studies on the neurophysiological mechanisms of acupuncture treatment, the analgesic effect of acupuncture treatment has been the most actively investigated research topic. Acupuncture treatment has the effect of controlling pain by stimulating the CNS through an intrinsic mechanism that mediates endogenous opioids [39]. Additionally, according to several reports, acupuncture treatment stimulates the vagus nerve and the nucleus of the tractus solitarius, which subsequently modulate the opioid receptors and project rostrally to the hypothalamus, amygdala, dorsal raphe nucleus, nucleus ambiguus, parabrachial nucleus, and thalamus in the amygdala, thus modulating any imbalance in the autonomic nervous system and making acupuncture effective for treating disorders of the autonomic nervous system, such as insomnia [40]. Thus, acupuncture stimulation influences the CNS by offering pain control and sedative effects.

Coherence is a variable used to measure a phase difference, thereby assessing the functional connectivity between cortices [15, 41]. Therefore, coherence offers additional information as compared to studies of the EEG power. If the EEG activity at two electrodes is synchronized, coherence values approach 1, whereas, if there is no synchronization, coherence values approach zero. Thus, higher levels of coherence between two electrodes suggest a strong functional connectivity between different cerebral regions [42].

This study found that, in the theta frequency band, coherence increased significantly during needle retention following* deqi* on the intrahemispheric temporooccipital regions. Furthermore, in the alpha band, coherence increased significantly during needle retention following* deqi* on the left intrahemispheric frontotemporal and front-occipital regions. Additionally, alpha coherence significantly increased on the interhemispheric parietal, temporal, and occipital regions. Similarly, a previous study on acupuncture treatment involving changes in coherence reported that theta coherence between the frontal and occipital regions increased immediately after* deqi,* as compared to before the acupuncture treatment and that electroacupuncture treatment increased occipital connectivity [32]. This report is inconsistent with the findings of the present study with regard to the area where theta coherence increased immediately after* deqi*. However, both studies converge in finding that theta coherence increased significantly immediately after* deqi* and coherence increased in the occipital region during electrical stimulation.

Although not significant in our results, theta coherence increased between the frontal and central regions during acupuncture treatment. This is in line with previous reports showing that phase-locked averaged pain-related neuronal responses occur at frequencies below 10 Hz [43, 44]. Meanwhile, in the frontofrontal and frontocentral regions, alpha coherence tended to decrease. This result conflicts with the results of a previous study that reported that alpha coherence increased in the frontocentral regions during pain stimulation [25], thus suggesting that acupuncture stimulation cannot cause the same changes as the perception of pain stimulation. Furthermore, the results of the present study, which involves increasing theta coherence in the frontal and central regions, do not cohere with those of previous studies reporting that there would be a negative relationship between anxiety and increasing theta activity at the frontal-midline [45, 46]. Therefore, we can suspect that it can be related to sedative effects of acupuncture.

Alpha coherence showed many significant changes in various regions. Phase coupling in the alpha frequency band is known to play an important role in the top-down processing [47–49]. Therefore, a significant increase in alpha coherence suggests that the effect of acupuncture would be driven not only by bottom-up mechanisms due to external stimuli, but also by the top-down processing. Additionally, our findings that both theta and alpha coherence tend to increase are consistent with previous reports that, in various meditation states, both theta and alpha coherence increase compared to other resting times [46, 50–52]. Therefore, this suggests that acupuncture treatment would be effective in anxious states.

However, our study has several limitations. First, due to the small sample size, our findings are difficult to generalize. Furthermore, other studies have reported that needle stimulation accompanied by sharp pain might induce different neurophysiological changes from* deqi *without sharp pain [6–8]. Thirdly, the participants in the present study reported a wide range of* deqi*. Therefore, in future research, such heterogeneity associated with* deqi* sensation should be controlled for.

## 5. Conclusion

Acupuncture stimulation of the HT7 accompanying* deqi* induces a specific change in the coherence of the theta and alpha bands. The findings of this study provide evidence that the effectiveness triggered by* deqi* is affected by the CNS, particularly, the forebrain. However, this result needs to be replicated by further studies that would involve larger cohorts categorized according to their reported* deqi *sensation levels.

## Supplementary Material

Supplement  1: coherence of theta and alpha among all electrodes before acupuncture and during retention of acupuncture needle.

## Figures and Tables

**Table 1:** Characteristics of participants and *deqi* sensation.

Index	Mean ± SD
Age (years)	21.33 ± 2.18

Gender (male/female)	5/4

*Total score of deqi*	12.00 ± 8.06

*Aching deqi*	6.56 ± 4.75

Deep ache	0.56 ± 0.73
Dull ache	1.22 ± 1.20
Uncomfortable	1.33 ± 1.00
Heavy	1.00 ± 1.00
Pressure	0.67 ± 0.87
Bruised	0.56 ± 0.73
Stinging	1.22 ± 0.97

*Tingling deqi*	5.44 ± 3.84

Tingling	0.67 ± 0.71
Warm	0.33 ± 0.71
Spreading	0.78 ± 0.83
Fading	1.44 ± 1.24
Numb	0.44 ± 0.53
Twinge	0.78 ± 0.83
Pricking	1.56 ± 0.53
Sharp	0.89 ± 0.60
Electric shock	0.33 ± 0.50
Throbbing	1.00 ± 0.87

SD, standard deviation.
