# Supplementary material for: Deqi Induction by HT7 Acupuncture Alters Theta and Alpha Band Coherence in Human Healthy Subjects
Source: Evid Based Complement Alternat Med. 2017 Apr 6;2017:7107136. doi: 10.1155/2017/7107136 (PMC5397730; doi:10.1155/2017/7107136)
Supplement: Supplementary file 1 — Supplement 1: coherence of theta and alpha among all electrodes before acupuncture and during retention of acupuncture needle. [file 7107136.f1.docx]

Supplement 1. Coherence of theta and alpha among all electrodes before acupuncture and during retention of acupuncture needle

|  | Theta | | | | |  | Alpha | | | | |
| --- | --- | --- | --- | --- | --- | --- | --- | --- | --- | --- | --- |
|  | before acupuncture | | during acupuncture | | p-value |  | before acupuncture | | during acupuncture | | p-value |
|  | mean | SD | mean | SD |  |  | mean | SD | mean | SD |  |
| FP1-FP2 | 64.77 | 19 | 70.02 | 13.22 | 0.23 |  | 91.28 | 3.11 | 91.57 | 7.17 | 0.87 |
| FP1-F7 | 59.1 | 13.69 | 60.85 | 10.32 | 0.61 |  | 87.58 | 5.08 | 86.44 | 11.34 | 0.6 |
| FP1-F3 | 63.44 | 15.1 | 67.74 | 8.72 | 0.24 |  | 90.52 | 2.65 | 90.6 | 6.31 | 0.98 |
| FP1-Fz | 60.5 | 13.18 | 63.81 | 10.12 | 0.35 |  | 89.93 | 3.85 | 89.24 | 6.86 | 0.67 |
| FP1-F4 | 49.83 | 14.87 | 53.65 | 9.42 | 0.31 |  | 84.27 | 4.91 | 83.63 | 9.22 | 0.76 |
| FP1-F8 | 37.67 | 13.9 | 39.98 | 9.96 | 0.38 |  | 76.91 | 6.52 | 75.93 | 14.80 | 0.62 |
| FP1-T3 | 30.23 | 10.92 | 31.65 | 9.63 | 0.52 |  | 51.11 | 14.2 | 53.38 | 22.61 | 0.4 |
| FP1-C3 | 38.44 | 9.1 | 41.78 | 7.91 | 0.25 |  | 65.1 | 10.22 | 66.68 | 18.92 | 0.56 |
| FP1-Cz | 37.51 | 9.1 | 39.69 | 7.71 | 0.44 |  | 73.3 | 7.85 | 71.72 | 16.09 | 0.51 |
| FP1-C4 | 29.76 | 9.28 | 30.84 | 7.61 | 0.61 |  | 55.39 | 9.46 | 54.59 | 18.68 | 0.77 |
| FP1-T4 | 16.98 | 10.58 | 16.64 | 8.37 | 0.91 |  | 31.58 | 10.13 | 30.86 | 18.03 | 0.57 |
| FP1-T5 | 6.88 | 6.83 | 6.94 | 7.27 | 0.47 |  | 14.96 | 11.05 | 19.87 | 13.87 | 0.03 |
| FP1-P3 | 18.19 | 8.15 | 17.68 | 9.41 | 0.68 |  | 15.49 | 11.69 | 21.6 | 12.34 | 0.08 |
| FP1-Pz | 19.53 | 7.54 | 19.25 | 8.02 | 0.92 |  | 24.96 | 12.86 | 27.25 | 13.94 | 0.52 |
| FP1-P4 | 13.45 | 6.34 | 12.82 | 7.89 | 0.59 |  | 10.44 | 9.7 | 18.08 | 10.88 | 0.08 |
| FP1-T6 | 2.91 | 3.17 | 3.44 | 3.35 | 0.33 |  | 21.41 | 14.89 | 24.42 | 18.68 | 0.48 |
| FP1-O1 | 2.92 | 2.44 | 2.72 | 3.38 | 0.43 |  | 17.54 | 14.71 | 21.73 | 17.20 | 0.08 |
| FP1-O2 | 2.28 | 1.65 | 2.2 | 2.25 | 0.42 |  | 16.66 | 16.69 | 20.26 | 18.86 | 0.11 |
| FP2-F7 | 37.09 | 12.83 | 39.25 | 12.65 | 0.51 |  | 77.94 | 8.06 | 76.55 | 17.50 | 0.45 |
| FP2-F3 | 53.03 | 11.17 | 57.05 | 10.29 | 0.3 |  | 85.99 | 3.8 | 85.32 | 11.79 | 0.58 |
| FP2-Fz | 62.06 | 9.32 | 65.65 | 11.80 | 0.35 |  | 90.66 | 3.06 | 89.47 | 9.12 | 0.3 |
| FP2-F4 | 62.48 | 13.06 | 67.45 | 10.85 | 0.23 |  | 90.47 | 2.47 | 89.73 | 8.39 | 0.6 |
| FP2-F8 | 62.63 | 11.63 | 64.11 | 9.55 | 0.6 |  | 87.77 | 2.96 | 86.83 | 10.42 | 0.51 |
| FP2-T3 | 17.79 | 9.31 | 19.36 | 9.73 | 0.48 |  | 42.74 | 14.85 | 45.16 | 22.03 | 0.41 |
| FP2-C3 | 31.96 | 8.8 | 35.78 | 9.90 | 0.24 |  | 60.41 | 11.63 | 61.98 | 20.40 | 0.56 |
| FP2-Cz | 38.94 | 7.29 | 42.13 | 9.73 | 0.35 |  | 74.01 | 6.38 | 72.46 | 17.25 | 0.43 |
| FP2-C4 | 38.64 | 8.99 | 40.81 | 8.87 | 0.45 |  | 62.05 | 9.17 | 61.13 | 19.08 | 0.72 |
| FP2-T4 | 28.73 | 11.26 | 30.56 | 8.91 | 0.41 |  | 40.23 | 10.71 | 39.08 | 18.88 | 0.51 |
| FP2-T5 | 4.2 | 5.37 | 4.52 | 5.99 | 0.83 |  | 14.43 | 11.37 | 19.21 | 14.07 | 0.03 |
| FP2-P3 | 15.71 | 7.78 | 15.65 | 9.43 | 0.92 |  | 13.81 | 10.73 | 20.11 | 12.18 | 0.04 |
| FP2-Pz | 20.15 | 7.36 | 20.46 | 8.99 | 0.84 |  | 25.19 | 12.81 | 27.87 | 14.65 | 0.48 |
| FP2-P4 | 17.63 | 6.39 | 16.9 | 9.05 | 0.57 |  | 12.87 | 11.42 | 20.45 | 12.29 | 0.11 |
| FP2-T6 | 5.25 | 3.92 | 5.44 | 4.49 | 0.9 |  | 20.97 | 13.43 | 24.52 | 17.75 | 0.37 |
| FP2-O1 | 2.5 | 2.18 | 2.32 | 3.25 | 0.56 |  | 17.75 | 14.29 | 21.5 | 17.03 | 0.13 |
| FP2-O2 | 2.61 | 1.9 | 2.37 | 2.37 | 0.29 |  | 16.17 | 15.76 | 20.08 | 18.45 | 0.13 |
| F7-F3 | 62.91 | 13.42 | 65.44 | 11.21 | 0.4 |  | 89.57 | 4.58 | 88.42 | 10.23 | 0.55 |
| F7-Fz | 46.83 | 12.16 | 48.77 | 11.68 | 0.43 |  | 82.33 | 7.49 | 80.51 | 14.70 | 0.36 |
| F7-F4 | 32.59 | 13.09 | 34.76 | 11.37 | 0.42 |  | 73.18 | 8.53 | 71.99 | 17.58 | 0.56 |
| F7-F8 | 18.59 | 11.13 | 18.1 | 10.94 | 1 |  | 63.23 | 11.15 | 62.35 | 21.46 | 0.56 |
| F7-T3 | 54.23 | 13.47 | 56.48 | 12.25 | 0.51 |  | 67.59 | 9.96 | 68.92 | 19.60 | 0.56 |
| F7-C3 | 46.74 | 10.82 | 49.71 | 11.97 | 0.23 |  | 72.35 | 7.34 | 72.81 | 17.73 | 0.89 |
| F7-Cz | 34.31 | 10.55 | 35.94 | 11.25 | 0.4 |  | 70.21 | 9.88 | 68.21 | 18.85 | 0.39 |
| F7-C4 | 22.29 | 10.64 | 22.52 | 10.42 | 0.81 |  | 48.28 | 9.77 | 47.94 | 19.85 | 0.8 |
| F7-T4 | 9.25 | 9.01 | 8.29 | 7.98 | 0.62 |  | 23.73 | 10.13 | 24.18 | 17.55 | 0.72 |
| F7-T5 | 14.76 | 10 | 14.82 | 12.19 | 0.56 |  | 15 | 8.95 | 20.76 | 11.98 | 0.01 |
| F7-P3 | 24.21 | 10.38 | 23.84 | 12.57 | 0.69 |  | 20.68 | 14.97 | 26.52 | 16.26 | 0.27 |
| F7-Pz | 20.55 | 8.81 | 20.03 | 10.15 | 0.73 |  | 26.71 | 14.59 | 28.79 | 16.56 | 0.57 |
| F7-P4 | 11.89 | 7.19 | 11 | 8.81 | 0.31 |  | 8.71 | 9.06 | 16.51 | 11.59 | 0.05 |
| F7-T6 | 2.07 | 2.86 | 2.71 | 3.52 | 0.26 |  | 20.02 | 13.93 | 23.76 | 17.87 | 0.35 |
| F7-O1 | 4.39 | 4.03 | 4.15 | 5.25 | 0.18 |  | 15.12 | 12.3 | 20.21 | 15.21 | 0.11 |
| F7-O2 | 2.64 | 2.33 | 2.3 | 2.90 | 0.12 |  | 14.97 | 15.08 | 18.97 | 17.08 | 0.04 |
| F3-Fz | 84.51 | 7.22 | 87.02 | 4.63 | 0.13 |  | 95.73 | 1.44 | 95.72 | 3.95 | 0.97 |
| F3-F4 | 64.48 | 11.86 | 67.81 | 6.74 | 0.25 |  | 87.34 | 3.46 | 87.28 | 9.37 | 0.92 |
| F3-F8 | 35.27 | 9.7 | 37.22 | 8.91 | 0.41 |  | 74.46 | 7.79 | 73.61 | 17.31 | 0.6 |
| F3-T3 | 47.92 | 11.18 | 50.18 | 9.25 | 0.3 |  | 62.73 | 10.6 | 64.8 | 19.87 | 0.32 |
| F3-C3 | 71.18 | 9.15 | 74.85 | 7.84 | 0.09 |  | 81.02 | 6.94 | 82.79 | 13.72 | 0.28 |
| F3-Cz | 68.82 | 7.54 | 70.72 | 7.66 | 0.28 |  | 86 | 4.56 | 85.04 | 12.56 | 0.36 |
| F3-C4 | 50.36 | 10 | 50.5 | 8.81 | 0.87 |  | 63.65 | 8.01 | 63.51 | 18.25 | 0.89 |
| F3-T4 | 23.31 | 11.18 | 23.14 | 10.01 | 0.95 |  | 34.01 | 10.73 | 34.09 | 18.93 | 0.7 |
| F3-T5 | 17.77 | 9.37 | 17.97 | 12.27 | 0.67 |  | 14.07 | 8.12 | 19.56 | 11.35 | 0.01 |
| F3-P3 | 39.55 | 10.09 | 38.86 | 14.01 | 0.56 |  | 24.76 | 15.75 | 30.89 | 16.83 | 0.26 |
| F3-Pz | 40.88 | 9.22 | 39.68 | 12.12 | 0.41 |  | 36.12 | 14.76 | 38.43 | 17.24 | 0.59 |
| F3-P4 | 28.7 | 8.67 | 26.93 | 12.09 | 0.29 |  | 14.69 | 11.94 | 22.36 | 13.95 | 0.1 |
| F3-T6 | 7.44 | 5.39 | 6.7 | 6.27 | 0.16 |  | 17.56 | 12.83 | 21.98 | 16.66 | 0.18 |
| F3-O1 | 8.81 | 5.35 | 7.89 | 6.81 | 0.17 |  | 13.85 | 10.8 | 18.76 | 13.80 | 0.06 |
| F3-O2 | 6.95 | 4.34 | 5.59 | 4.84 | 0.06 |  | 13.17 | 13.38 | 17.23 | 15.62 | 0.07 |
| Fz-F4 | 81.37 | 11.82 | 84.41 | 5.76 | 0.25 |  | 94.94 | 1.83 | 94.93 | 4.16 | 0.98 |
| Fz-F8 | 48 | 7.65 | 51.05 | 8.35 | 0.13 |  | 82.69 | 6.04 | 81.36 | 13.97 | 0.43 |
| Fz-T3 | 35.21 | 10.15 | 36.43 | 10.15 | 0.46 |  | 53.44 | 12.94 | 55.55 | 21.79 | 0.43 |
| Fz-C3 | 65.63 | 8.9 | 68.15 | 8.87 | 0.07 |  | 75.55 | 9.81 | 77.71 | 16.67 | 0.31 |
| Fz-Cz | 77.95 | 6.2 | 78.5 | 7.51 | 0.33 |  | 89.1 | 3.67 | 88.79 | 11.03 | 0.71 |
| Fz-C4 | 62.87 | 10.39 | 63.93 | 9.26 | 0.51 |  | 71.51 | 8.06 | 71.56 | 17.01 | 0.97 |
| Fz-T4 | 32.83 | 11.77 | 33.89 | 10.91 | 0.47 |  | 41.38 | 11.2 | 40.83 | 19.23 | 0.61 |
| Fz-T5 | 13.36 | 8.45 | 12.93 | 10.20 | 0.22 |  | 13.34 | 8.2 | 18.34 | 11.46 | 0.02 |
| Fz-P3 | 36.74 | 10.35 | 35.28 | 14.11 | 0.13 |  | 22.08 | 15.26 | 28.66 | 16.19 | 0.18 |
| Fz-Pz | 43.41 | 10.07 | 41.86 | 13.09 | 0.24 |  | 36.77 | 14.95 | 39.78 | 17.35 | 0.51 |
| Fz-P4 | 34.68 | 9.05 | 32.63 | 13.39 | 0.21 |  | 17.68 | 13.73 | 25.44 | 14.87 | 0.12 |
| Fz-T6 | 10.34 | 6.49 | 9.77 | 8.13 | 0.22 |  | 18.18 | 12.15 | 22.31 | 16.15 | 0.25 |
| Fz-O1 | 8.17 | 4.93 | 6.94 | 5.88 | 0.14 |  | 14.49 | 11.36 | 18.67 | 13.98 | 0.09 |
| Fz-O2 | 7.65 | 4.44 | 6.36 | 5.11 | 0.1 |  | 13.61 | 13.24 | 17.39 | 15.56 | 0.1 |
| F4-F8 | 62.75 | 12.98 | 67.94 | 7.05 | 0.06 |  | 89.88 | 3.63 | 89.37 | 8.04 | 0.72 |
| F4-T3 | 23.21 | 9.99 | 24.92 | 9.73 | 0.36 |  | 44.68 | 13.15 | 47.5 | 21.17 | 0.38 |
| F4-C3 | 49.66 | 8.79 | 53.35 | 7.75 | 0.13 |  | 67.3 | 10.48 | 70.06 | 17.85 | 0.3 |
| F4-Cz | 66.21 | 10.38 | 68.79 | 7.40 | 0.28 |  | 85.56 | 4.03 | 85.54 | 11.86 | 0.96 |
| F4-C4 | 66.92 | 14.92 | 69.94 | 9.11 | 0.2 |  | 78.08 | 7.51 | 78.47 | 13.68 | 0.87 |
| F4-T4 | 43.31 | 16.13 | 47.21 | 10.83 | 0.15 |  | 51.27 | 11.46 | 50.07 | 17.53 | 0.56 |
| F4-T5 | 8.46 | 6.94 | 8.45 | 8.00 | 0.4 |  | 12.18 | 8.26 | 17.09 | 11.45 | 0.03 |
| F4-P3 | 28.48 | 9.13 | 28.2 | 12.07 | 0.73 |  | 19.09 | 13.34 | 26.09 | 15.94 | 0.15 |
| F4-Pz | 37.6 | 9.58 | 37.96 | 11.26 | 0.88 |  | 35.34 | 14.06 | 39.23 | 17.29 | 0.42 |
| F4-P4 | 35.53 | 9.18 | 35.16 | 12.88 | 0.75 |  | 20.74 | 14.8 | 28.35 | 16.66 | 0.19 |
| F4-T6 | 12.8 | 6.74 | 13.07 | 9.67 | 0.5 |  | 18.4 | 10.02 | 22.41 | 14.86 | 0.35 |
| F4-O1 | 6.43 | 4.24 | 5.58 | 4.96 | 0.22 |  | 14.26 | 10.59 | 18.08 | 13.51 | 0.13 |
| F4-O2 | 7.41 | 3.88 | 6.45 | 5.04 | 0.15 |  | 13.29 | 11.55 | 17.18 | 14.65 | 0.11 |
| F8-T3 | 9.46 | 8.45 | 10.1 | 8.22 | 0.5 |  | 33.8 | 14.11 | 36.66 | 20.20 | 0.44 |
| F8-C3 | 23.25 | 8.77 | 26.15 | 9.15 | 0.15 |  | 53.47 | 11.52 | 55.54 | 20.99 | 0.58 |
| F8-Cz | 34.53 | 8.5 | 37.77 | 9.50 | 0.15 |  | 71.49 | 7.45 | 70.4 | 18.59 | 0.59 |
| F8-C4 | 45.01 | 11.71 | 50.12 | 8.40 | 0.06 |  | 71.86 | 8.7 | 71.15 | 16.64 | 0.76 |
| F8-T4 | 48.27 | 18.5 | 54.94 | 11.09 | 0.08 |  | 58.29 | 12.02 | 57.28 | 16.87 | 0.56 |
| F8-T5 | 2.48 | 4.46 | 2.57 | 5.04 | 0.68 |  | 13.07 | 10.45 | 17.43 | 12.39 | 0.07 |
| F8-P3 | 11.89 | 8.29 | 12.29 | 9.03 | 0.65 |  | 12.87 | 9.88 | 19.22 | 13.70 | 0.1 |
| F8-Pz | 18.64 | 8.65 | 19.8 | 9.53 | 0.42 |  | 27.05 | 13.04 | 30.13 | 16.53 | 0.5 |
| F8-P4 | 20.94 | 8.65 | 21.96 | 10.70 | 0.56 |  | 18.82 | 15.18 | 25.56 | 16.75 | 0.24 |
| F8-T6 | 9.28 | 6.42 | 10.27 | 8.17 | 0.81 |  | 20.11 | 9.44 | 23.6 | 14.41 | 0.49 |
| F8-O1 | 2.36 | 2.16 | 2.13 | 3.20 | 0.55 |  | 16.26 | 12.53 | 19.73 | 15.06 | 0.14 |
| F8-O2 | 3.34 | 2.55 | 3.04 | 2.99 | 0.25 |  | 14.37 | 11.53 | 18.34 | 15.58 | 0.16 |
| T3-C3 | 60.6 | 13.33 | 63.05 | 10.74 | 0.23 |  | 78.81 | 6.54 | 79.13 | 15.34 | 0.86 |
| T3-Cz | 38.44 | 10.14 | 39.81 | 10.26 | 0.34 |  | 58.02 | 11.23 | 58.97 | 19.10 | 0.73 |
| T3-C4 | 24.52 | 10.03 | 24.54 | 9.92 | 0.9 |  | 37.76 | 10.07 | 40.13 | 17.43 | 0.51 |
| T3-T4 | 8.91 | 8.59 | 8.18 | 7.16 | 0.73 |  | 15.52 | 7.61 | 17.77 | 12.26 | 0.61 |
| T3-T5 | 43.62 | 12.56 | 46.04 | 15.60 | 0.22 |  | 28.61 | 20.16 | 31.7 | 16.77 | 0.32 |
| T3-P3 | 48.38 | 13.23 | 49.71 | 13.25 | 0.19 |  | 48.76 | 17.33 | 49.89 | 17.49 | 0.85 |
| T3-Pz | 35.03 | 10.35 | 35.07 | 11.36 | 0.83 |  | 40.41 | 10.94 | 41.56 | 15.38 | 0.76 |
| T3-P4 | 20.07 | 8.81 | 19.39 | 9.98 | 0.56 |  | 13.11 | 8.37 | 17.84 | 12.13 | 0.17 |
| T3-T6 | 4.19 | 4.34 | 4.09 | 5.25 | 0.24 |  | 10 | 9.12 | 13.61 | 10.79 | 0.16 |
| T3-O1 | 17.58 | 7.65 | 17.32 | 9.61 | 0.62 |  | 12.24 | 10.19 | 16.2 | 9.28 | 0.03 |
| T3-O2 | 8.18 | 4.53 | 7.54 | 5.99 | 0.24 |  | 6.47 | 7.29 | 10.19 | 7.72 | 0.04 |
| C3-Cz | 79.4 | 6.82 | 82.07 | 4.29 | 0.08 |  | 85.71 | 6.73 | 86.97 | 7.52 | 0.33 |
| C3-C4 | 56.8 | 12.06 | 58.48 | 8.31 | 0.42 |  | 63.07 | 9.5 | 65.25 | 14.38 | 0.46 |
| C3-T4 | 24.07 | 11.91 | 24.73 | 10.61 | 0.54 |  | 31.37 | 9.52 | 33.57 | 17.35 | 0.68 |
| C3-T5 | 39.3 | 9.96 | 39.15 | 13.66 | 0.73 |  | 20.11 | 15.77 | 24.97 | 13.16 | 0.13 |
| C3-P3 | 70.21 | 11.45 | 70.25 | 11.78 | 0.94 |  | 53.45 | 18.2 | 56.47 | 16.83 | 0.5 |
| C3-Pz | 65.85 | 9.88 | 66.03 | 9.56 | 0.85 |  | 58.96 | 11.81 | 60.63 | 11.45 | 0.66 |
| C3-P4 | 45.76 | 9.97 | 44.93 | 12.36 | 0.62 |  | 26.28 | 13.8 | 31.84 | 15.78 | 0.21 |
| C3-T6 | 14.07 | 7.5 | 13.26 | 9.73 | 0.26 |  | 12 | 7.67 | 16.86 | 11.78 | 0.13 |
| C3-O1 | 24.5 | 8.13 | 22.52 | 9.77 | 0.17 |  | 12.23 | 9.54 | 17.03 | 8.79 | 0.04 |
| C3-O2 | 17.54 | 6.41 | 15.64 | 7.69 | 0.14 |  | 9.08 | 6.39 | 13.22 | 8.07 | 0.09 |
| Cz-C4 | 76.24 | 10.31 | 78.03 | 6.57 | 0.32 |  | 83.15 | 4.92 | 83.67 | 9.13 | 0.78 |
| Cz-T4 | 35.96 | 12.94 | 37.92 | 11.75 | 0.26 |  | 46.94 | 10.6 | 46.75 | 18.12 | 0.74 |
| Cz-T5 | 24.31 | 8.87 | 24.01 | 11.88 | 0.5 |  | 12.69 | 9.67 | 17.76 | 11.03 | 0.03 |
| Cz-P3 | 59.02 | 9.02 | 58.45 | 12.15 | 0.51 |  | 39.42 | 19.1 | 44.9 | 18.43 | 0.2 |
| Cz-Pz | 70.68 | 7.35 | 70 | 8.75 | 0.48 |  | 61.94 | 15.53 | 64.17 | 12.78 | 0.55 |
| Cz-P4 | 57.04 | 7.49 | 55.54 | 11.50 | 0.37 |  | 34.29 | 18.26 | 39.97 | 17.33 | 0.27 |
| Cz-T6 | 19.89 | 8.91 | 19.01 | 11.77 | 0.33 |  | 15.01 | 8.07 | 19.38 | 11.77 | 0.28 |
| Cz-O1 | 19.36 | 7.76 | 17.83 | 9.28 | 0.23 |  | 11.2 | 7.25 | 15.51 | 9.00 | 0.13 |
| Cz-O2 | 19.1 | 6.88 | 17.24 | 8.16 | 0.17 |  | 11.19 | 7.28 | 14.72 | 8.14 | 0.16 |
| C4-T4 | 54.75 | 20.5 | 60.42 | 13.34 | 0.08 |  | 71.57 | 10.79 | 70.28 | 13.63 | 0.51 |
| C4-T5 | 16.86 | 8.35 | 16.43 | 10.02 | 0.4 |  | 9.61 | 7 | 14.54 | 10.78 | 0.06 |
| C4-P3 | 45.95 | 11.89 | 45.78 | 12.62 | 0.99 |  | 32.8 | 14.8 | 39.09 | 18.07 | 0.11 |
| C4-Pz | 61.31 | 12.86 | 62.26 | 11.38 | 0.47 |  | 57.87 | 14.54 | 61.94 | 15.03 | 0.32 |
| C4-P4 | 65.42 | 13.75 | 66.69 | 12.29 | 0.47 |  | 50.84 | 18.52 | 55.48 | 17.68 | 0.4 |
| C4-T6 | 32.82 | 11.57 | 32.73 | 14.19 | 0.76 |  | 21.46 | 13.99 | 25.46 | 13.30 | 0.49 |
| C4-O1 | 17.14 | 8.13 | 15.74 | 8.77 | 0.29 |  | 10.08 | 6.6 | 14.64 | 10.66 | 0.21 |
| C4-O2 | 22.53 | 7.61 | 20.78 | 7.95 | 0.32 |  | 12.37 | 8.68 | 17.22 | 9.22 | 0.11 |
| T4-T5 | 5.89 | 6.42 | 5.51 | 6.26 | 0.54 |  | 6.37 | 5.08 | 9.4 | 8.95 | 0.25 |
| T4-P3 | 20.24 | 12 | 20.2 | 10.85 | 0.74 |  | 16.81 | 8.01 | 22.39 | 15.17 | 0.1 |
| T4-Pz | 31.23 | 13.94 | 31.94 | 12.53 | 0.48 |  | 33.5 | 8.14 | 37.75 | 16.50 | 0.35 |
| T4-P4 | 43.52 | 17.28 | 47.18 | 14.58 | 0.08 |  | 49.17 | 14.4 | 52.54 | 18.06 | 0.47 |
| T4-T6 | 34.75 | 14.84 | 40.35 | 14.68 | 0.02 |  | 34.4 | 20.8 | 37.07 | 15.97 | 0.48 |
| T4-O1 | 8.06 | 6.92 | 7.39 | 5.69 | 0.74 |  | 8.35 | 3.69 | 11.28 | 9.15 | 0.54 |
| T4-O2 | 15.43 | 8.56 | 15.64 | 7.32 | 0.58 |  | 13.78 | 10.02 | 17.72 | 8.75 | 0.2 |
| T5-P3 | 65.7 | 7.08 | 69.12 | 7.33 | 0.03 |  | 63.17 | 13.24 | 67.39 | 9.11 | 0.21 |
| T5-Pz | 42.16 | 8.14 | 43.17 | 11.26 | 0.6 |  | 28.32 | 16.54 | 34.52 | 13.33 | 0.09 |
| T5-P4 | 28.3 | 6.43 | 30.02 | 10.01 | 0.51 |  | 20.82 | 12.62 | 27.17 | 13.20 | 0.03 |
| T5-T6 | 14 | 4.61 | 15.42 | 6.53 | 0.55 |  | 18.98 | 19.65 | 23.18 | 16.96 | 0.03 |
| T5-O1 | 60.12 | 9.37 | 64.25 | 8.22 | 0.05 |  | 72.84 | 7.94 | 75.31 | 10.14 | 0.27 |
| T5-O2 | 29.2 | 5.95 | 32.25 | 6.99 | 0.17 |  | 29.06 | 18.89 | 34.13 | 16.27 | 0.04 |
| P3-Pz | 80.08 | 8.14 | 81.57 | 7.53 | 0.09 |  | 77.58 | 9.34 | 80.86 | 9.08 | 0.01 |
| P3-P4 | 59.14 | 8.63 | 60.39 | 9.84 | 0.45 |  | 49.5 | 13.24 | 54.83 | 16.75 | 0.01 |
| P3-T6 | 24.62 | 7.83 | 25.8 | 10.24 | 0.71 |  | 17.15 | 14.83 | 23.99 | 14.27 | 0.04 |
| P3-O1 | 58.56 | 7.29 | 60.34 | 7.25 | 0.35 |  | 53.04 | 16.96 | 58.77 | 7.53 | 0.21 |
| P3-O2 | 40.73 | 6.57 | 41.81 | 6.89 | 0.64 |  | 27.93 | 16.12 | 33.35 | 12.21 | 0.1 |
| Pz-P4 | 77.49 | 9.32 | 78.14 | 9.50 | 0.6 |  | 70.78 | 14.11 | 73.95 | 14.38 | 0.04 |
| Pz-T6 | 34.34 | 10.94 | 34.66 | 13.90 | 0.88 |  | 22.57 | 17.41 | 27.97 | 15.78 | 0.07 |
| Pz-O1 | 48.03 | 8.63 | 48.22 | 10.30 | 0.99 |  | 30.7 | 20.45 | 37.09 | 11.88 | 0.12 |
| Pz-O2 | 47.74 | 8.08 | 47.6 | 9.01 | 0.87 |  | 31.43 | 19.28 | 34.72 | 13.72 | 0.19 |
| P4-T6 | 59.76 | 11.34 | 63.22 | 11.11 | 0.06 |  | 59.42 | 14.95 | 64.33 | 9.60 | 0.1 |
| P4-O1 | 40.27 | 9.73 | 41.87 | 9.21 | 0.45 |  | 32.2 | 18.12 | 40.15 | 16.35 | 0.01 |
| P4-O2 | 59.04 | 7.78 | 60.95 | 6.22 | 0.26 |  | 54.85 | 13.08 | 60.54 | 10.58 | 0.11 |
| T6-O1 | 26.2 | 6.88 | 28.73 | 7.57 | 0.26 |  | 32.12 | 21.17 | 38.15 | 20.76 | 0.01 |
| T6-O2 | 55.17 | 10.22 | 59.17 | 7.98 | 0.02 |  | 62.36 | 19.79 | 66.85 | 14.82 | 0.04 |
| O1-O2 | 59.67 | 9.82 | 62.79 | 8.33 | 0.34 |  | 61 | 18.04 | 65.27 | 17.27 | 0.08 |
